# Small-Molecule Tyrosinase Inhibitors for Treatment of Hyperpigmentation

**DOI:** 10.3390/molecules30040788

**Published:** 2025-02-08

**Authors:** Xinhua Ni, Xinyu Luo, Xiaoying Jiang, Wenchao Chen, Renren Bai

**Affiliations:** 1 School of Pharmacy, Hangzhou Normal University, Hangzhou 311121, China; 2 Key Laboratory of Elemene Class Anti-Tumor Chinese Medicines, Engineering Laboratory of Development and Application of Traditional Chinese Medicines, Collaborative Innovation Center of Traditional Chinese Medicines of Zhejiang Province, Hangzhou Normal University, Hangzhou 311121, China

**Keywords:** melanin, hyperpigmentation, tyrosinase inhibitor, structure–function relationships (SARs)

## Abstract

Increasing attention is being focused on skin health currently, especially the excessive deposition of melanin in the skin. Tyrosinase, the rate-limiting enzyme in melanin biosynthesis, is a crucial enzyme in melanin synthesis. However, existing tyrosinase inhibitors pose some degree of toxicity to humans. Therefore, the development of more efficient and low-toxicity tyrosinase inhibitors is urgently needed. This review briefly depicts the melanin biosynthesis process and the crystal structure and catalytic mechanism of tyrosinase. The latest research progress regarding small-molecule tyrosinase inhibitors is also reviewed. Moreover, the structure–function relationships are analyzed and summarized. This is expected to provide new and more scientific insights to enable researchers to explore safer and more potent tyrosinase inhibitors.

## 1. Introduction

Melanin, a natural pigment, mainly including eumelanin, pheomelanin, and neuromelanin, performs an instrumental function in the human pigment composition system [1]. Eumelanin and pheomelanin ultimately contribute to the color of human skin, hair, and eyes through providing cells with varying degrees of pigmentation [1,2]. Melanin demonstrates reducing capabilities and can reversibly bind with metal ions, thereby enabling it to scavenge free radicals and exhibit antioxidant effects [3]. Additionally, melanin protects the skin from harmful bright radiation (UVR) [4,5]. However, excessive melanin production and accumulation can cause severe skin pigmentation issues, including freckles [6], pigmented acne [7], chloasma [6,7], and age spots [8] and may even increase the risk of malignant melanoma [5,9]. In Asian populations, 40% of women and 20% of men are impacted by melasma [10]. Furthermore, approximately 90% of melanoma cases are cutaneous melanoma, making it the most common subtype of the disease [11]. Therefore, abnormal melanin production and excessive accumulation can negatively affect psychological, emotional, and physical health [12].

The essential and prevalent strategies for the treatment of hypermelanosis are the suppression of melanin synthesis and the reduction of the melanin concentration [5,13,14]. Melanin synthesis is involved in the stimulation of the melanocortin 1 receptor (MC-1R) via *α*-melanocyte-stimulating hormone (*α*-MSH), which further activates microphthalmia-associated transcription factor (MITF) and controls the gene expression of tyrosinase (TYR), ultimately promoting melanin synthesis [13,15,16]. Meanwhile, the melanin biosynthesis pathway begins with the oxidation of monophenols, including *_L_*-tyrosine or diphenol-like *_L_*-dihydroxyphenylalanine (*_L_*-DOPA), to form dopaquinone (DQ) [17]. This reaction, mediated by TYR, is the rate-limiting step in the overall melanin synthesis pathway [18,19]. As the melanin synthesis step following DQ is mainly a non-enzymatic reaction, TYR plays a pivotal role as the rate-limiting enzyme [20]. Therefore, the inhibition of TYR activity decreases melanogenesis and thus regulates excessive melanin deposition [21,22].

Moreover, TYR has been considered one of the hypothetical therapeutic targets regarding melanoma due to its significant contribution to melanogenesis [19,23]. In stage I melanoma, the presence of melanin may offer benefits to patients; however, in advanced melanoma, melanin synthesis can negatively impact treatment outcomes, resulting in diminished overall survival and disease-free survival rates [24]. Moreover, melanin synthesis during radiotherapy and chemotherapy can contribute to heightened resistance in melanoma [25,26,27]. The administration of inhibitors as an adjuvant therapy to sensitize melanoma cells may effectively reduce their multidrug resistance, thereby improving patients’ prognosis [28,29]. Brożyna et al. analyzed the impact of melanogenesis in patients with phase III and IV melanomas, as well as investigating the melanin content of metastatic melanomas and its effect on radiotherapy. It was shown that melanogenesis shortened the survival of patients with metastatic melanoma and attenuated the effects of radiotherapy [25,30]. In parallel, Slominski et al. evaluated the effects of TYR inhibitors, including *N*-phenylthiourea (PTU) and *_D_*-penicillamine, on the cyclophosphamide inhibition of human melanoma cells (SKMEL-188). It was revealed that TYR inhibitors could sensitize melanoma cells to the cytotoxic effects of cyclophosphamide and could enhance the activity of IL-2, reducing the effective cell proliferation inhibitory concentration from the original 10^−3^ M to 10^−6^ M [31]. Additionally, PTU and *_D_*-penicillamine can also enhance the sensitivity of melanoma cells to γ-ray irradiation at doses between 2 and 15 Gy by inhibiting melanin production. Findings indicate that non-pigmented cells, following depigmentation treatment with PTU or *_D_*-penicillamine, display significantly lower resistance to gamma-ray radiation than pigmented cells. This is particularly pronounced at the 15 Gy dose, where the growth inhibition is most pronounced and the survival rate of pigmented cells is approximately 40% higher than that of non-pigmented cells, with the inhibitory effect being time-dependent [28]. Therefore, TYR inhibitors can also be serve as an adjuvant therapy for melanoma [32].

Multiple TYR inhibitors, including tretinoin, arbutin, hydroquinone (HQ), ellagic acid, azelaic acid, *_L_*-ascorbic acid, and tranexamic acid, have been applied as anti-pigmentation agents [33]. However, they also have certain drawbacks, including potential toxicity and low tissue permeability, resulting in being unsuccessful for clinical use. Therefore, it is crucial to pursue the discovery of novel TYR inhibitors with more potent pharmacologic activity, better drug-like properties, and fewer side effects [33,34].

At present, several reviews have offered a comprehensive and systematic overview of TYR inhibitors before 2023, elaborating on their findings in detail [19,35,36,37]. However, TYR inhibitors introduced from 2024 onward have not yet been considered due to temporal limitations. This review briefly introduces the melanin biosynthesis pathway and examines the structural features and catalytic mechanisms of TYR. More importantly, we comprehensively review promising TYR inhibitors in the drug discovery phase and their structure–activity relationships (SARs) over the past five years, including 2025, providing practical information and promising insights.

## 2. Melanin: Related Pathways and Biosynthesis Process

The formation of melanin within organisms involves a range of intricate enzyme-catalyzed reactions mediated by three key enzymes: TYR, TYR-related protein 1 (TYRP-1), and TYR-related protein 2 (TYRP-2). TYR, a type III copper-containing oxidoreductase, participates in the initial stages of melanin synthesis [38]. Both TYRP-1 and TYRP-2, with two zinc ions in their active sites, also play vital roles in melanin biosynthesis. TYRP-2 has also been evidenced to possess isomerase activity [39,40].

Under physiological conditions, melanin is synthesized in melanosomes, limited to melanocytes [41]. Melanin synthesis requires the co-regulation of multiple intracellular signaling pathways, including the mitogen-activated protein kinase (MAPK) pathway [42], cyclic adenosine monophosphate (cAMP)/protein kinase A (PKA) pathway [43], Wnt/β-catenin pathway [44], phosphatidylinositol-3-kinase (PI3K)/Akt pathway [45], and nitric oxide (*NO*)-related pathway [46]. MITF serves as a master regulator of these five critical signaling pathways, thereby establishing a complex network that coordinates melanin production [16,47,48,49].

**Figure 1:**
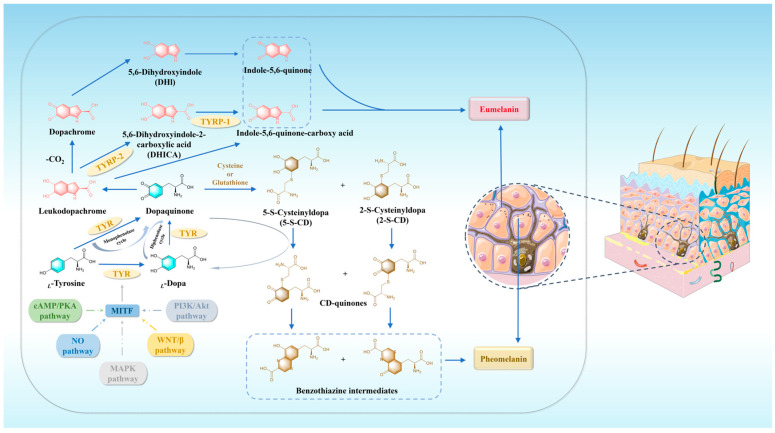
The biosynthesis process of melanin.

*_L_*-Tyrosine is oxidized through the monophenolase cycle to produce DQ, a crucial substrate for eumelanin and pheomelanin production. This oxidation process also initiates melanin synthesis [50]. *_L_*-Tyrosine can also be hydroxylated to form *_L_*-DOPA by hydroxylation and subsequently further oxidized via the diphenolase cycle to produce DQ. The formation of DQ is widely acknowledged as the phase that limits the rate of melanin biosynthesis, since subsequent reactions can occur spontaneously at the physiological pH. In the presence of a small amount of TYR, DQ reacts with cysteine or glutathione to form cysteinyl dopa or glutathione dopa. These compounds undergo a series of redox reactions to form benzothiazine intermediates, which further undergo a complex polymerization reaction, ultimately resulting in the formation of pheomelanin. In addition, an excess of TYR can lead to the self-cyclization of DQ, resulting in the formation of leukodopachrome, consequently being converted to dopachrome. Dopachrome undergoes decarboxylation to produce 5,6-dihydroxyindole (DHI), and further oxidation by TYR yields indole-5,6-quinone. TYRP-2 catalyzes the conversion of dopachrome to 5,6-dihydroxyindole carboxylic acid (DHICA) [51]. Subsequently, the oxidation of DHICA and leukodopachrome results in the formation of indole-5,6-quinone carboxylic acid, which finally polymerizes with indole-5,6-quinone to form eumelanin (Figure 1) [52].

## 3. TYR: Structural Features and Catalytic Mechanism

### 3.1. The Structure of TYR

**Figure 2:**
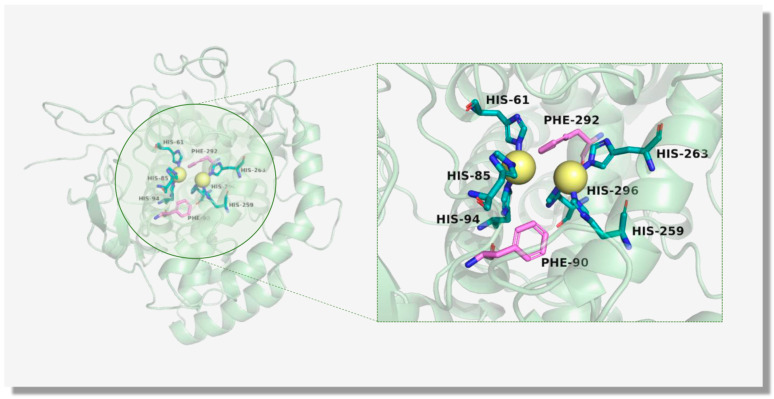
The active core of mTYR (PDB ID: 2Y9X) is illustrated via a cartoon model, containing two copper ions accompanied by histidine residues.

The TYR structure can be divided into three domains from a structural standpoint: the central domain, the *N*-terminal domain, and the transmembrane domain. The active core of TYR, the central structural domain, which includes two copper-binding sites known as Cu(A) and Cu(B), is conserved in all TYRs across various sources, representing the sole conserved segment of the enzyme [53]. Each of the two copper atoms connected by an oxygen atom interacts strongly with three specific histidine residues (Figure 2) [54]. In parallel, in the active site, the establishment of thioether bonds between cysteine and histidine residues, as well as hydrogen bonding between the *N* atom on the histidine and the *O* atom of the peptide carbonyl, is conducive to the maintenance of the ordered geometry of the active core [39,51,55,56].

It was not until 2006 that Matoba et al. successfully determined the inaugural crystal structure of *Streptomyces castaneoglobisporu* TYR complexed with globular proteins (ORF378) in *Escherichia coli*, and they discovered that the active site of TYR, integrated with copper ions, exhibits flexibility throughout the catalytic process [56]. Based on molecular and biochemical techniques, Ismaya et al. elucidated the crystal structure of *Agaricus bisporus* TYR (mTYR) in 2011 [57]. mTYR was found to be an H_2_L_2_ tetramer structure with a molecular weight of 120 kDa. The H subunit, comprising 13 *α*-helices, 8 short *β*-strands, and multiple loops, weighs about 43 kDa. The L subunit, containing 12 reverse-parallel *β*-strands containing 150 amino acids, weighs 14 kDa. Importantly, it is highly similar to the enzyme core region of TYR and is more readily available and cost-effective [57]. Therefore, mTYR is frequently selected as the target enzyme in new inhibitor discovery [56,57,58].

### 3.2. The Catalytic Mechanism of TYR

The deoxy form (*E_deoxy_*), oxygen form (*E_oxy_*), and methoxy form (*E_met_*) are the three distinguished forms of TYR involved in catalytic reactions [54,59]. It exhibits dual catalytic activity, functioning as both a monophenolase and a diphenolase on phenolic compounds [35,51,60].

The monophenolase *E_oxy_* complex (*E_oxy_*M) is generated during the monophenolase cycle through the interaction between a monophenol substrate and a vertically oriented copper ion located within the *E_oxy_* active site. The rate of this reaction is termed monophenolase activity [61,62]. Subsequently, the monophenol substrate forms the *ortho*-electrophilically substituted diphenolase *E_met_* complex (*E_met_*D). The reactive *E_met_*D is cleaved to produce *ortho*-quinone and *E_deoxy_* directly, while *E_oxy_* is reduced to *E_deoxy_*. Finally, oxygen interacts with *E_deoxy_*, regenerating *E_oxy_* [35]. In particular, with *E_met_* lacking the capability to bind the oxygen in the monophenol cycle reaction, it undergoes a very slow oxidation reaction when *E_met_* in its natural state encounters a monophenol substrate, preventing the monophenol reaction from proceeding properly [61,63].

Within the process of diphenolase activity, *E_oxy_* interacts with catechol and oxidizes it to *ortho*-benzoquinone, and then *E_met_* is obtained, and the rate of oxidation of catechol to *ortho*-benzoquinone is the diphenolase activity [64]. It has been demonstrated that monophenol and catechol are subjected to competition for the active site of *E_met_*, and the combination of monophenol and *E_met_* will produce an inactive complex (*E_met_*M), which will drop out of the catalytic cycle of bisphenol enzymes. Moreover, with the assistance of oxygen, *E_deoxy_* can be converted to E_oxy_, which is able to oxidize bisphenol as well as monophenol [52]. The difference is that monophenol has a greater tendency to form complexes with Cu(A), whereas bisphenol is initially more inclined to bind to Cu(B) [50]. Catechol can eventually undergo oxidation to become *ortho*-benzoquinone, resulting in the transition of TYR from *E_met_* to *E_deoxy_* [65,66].

The catalytic mechanism of TYR reveals its essential and important contribution to melanin biosynthesis.

## 4. Advanced Research Progress in TYR Inhibitors: Natural Products and Small-Molecule Compounds

### 4.1. TYR Inhibitors from Natural Products

In recent years, TYR inhibitors from natural sources have garnered increasing attention [67]. Many researchers opt to identify inhibitors from natural sources because of their reduced toxicity and improved bioavailability. Certain natural products demonstrate remarkably weak cytotoxicity against the B16F10 cell line, with an LD_50_ value reaching 137.8 ± 1.2 μM, which significantly exceeds their IC_50_ values for enzyme inhibition [68]. Natural products offer a wide range of TYR inhibitors, including arbutin [69], resveratrol [70], and galangin [71]. The structures, activity, sources, and additional information about representative natural product-based tyrosinase inhibitors are summarized in Table 1. Based on their structural features, natural TYR inhibitors can be categorized into polyphenols, flavonoids, stilbenes, flavonolignans, etc.

### 4.2. Small-Molecule TYR Inhibitors in the Drug Discovery Phase

Small-molecule TYR inhibitors with potent inhibitory activity that were reported from 2020 to the present were comprehensively reviewed. In addition to the relevant structures and enzyme-inhibitory activity, the detailed SARs of a representative chemical series of TYR inhibitors were summarized and analyzed, including azoles, thioureas, amides, cinnamic acid, and other types.

#### 4.2.1. Azole Derivatives

##### Triazole Derivatives

**Figure 3:**
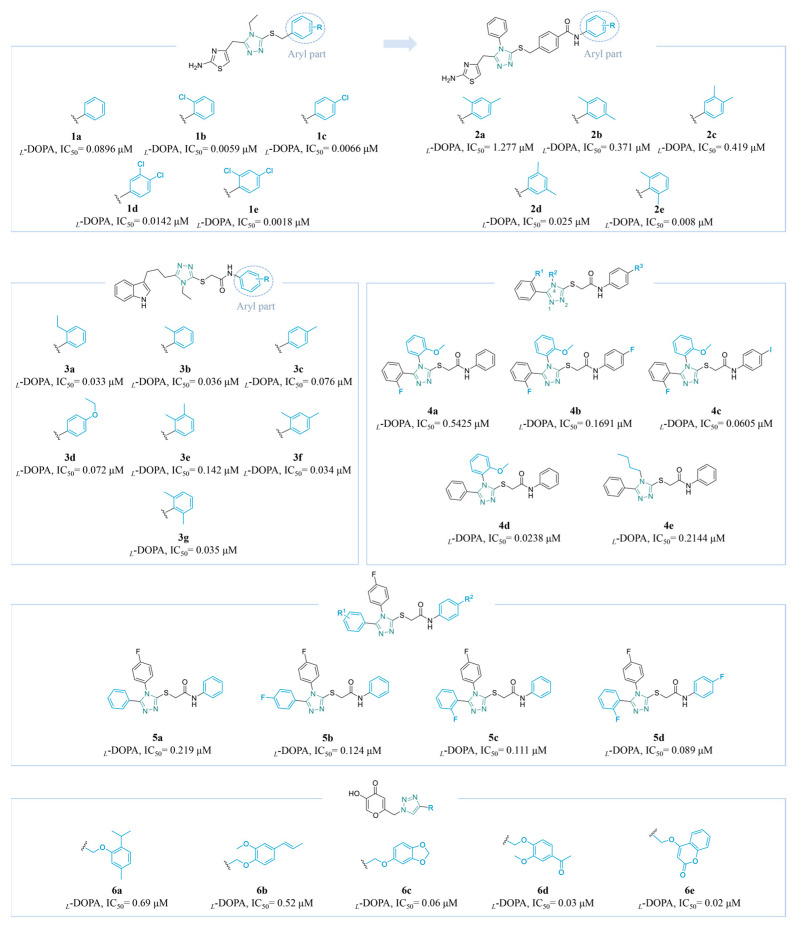
Chemical structures of TYR inhibitors **1a**–**6e** with potent anti-TYR activity.

Abbasi and colleagues synthesized a range of aralkylated hybrids of 2-aminothiazole-ethyltriazole (**1a**–**e**) (Figure 3) and assessed their anti-TYR activity. Derivatives with *ortho-* and *para-*chloro or di-chloro groups in the benzylic moiety generally exhibited superior inhibitory potential (**1a**: IC_50_ = 0.0896 μM; **1b**: IC_50_ = 0.0059 μM; **1c**: IC_50_ = 0.0066 μM; **1d**: IC_50_ = 0.0142 μM). Furthermore, it was found that the chloro groups at the 2- and 4-positions in the benzylic part were suitable for the inhibition of TYR (**1e**: IC_50_ = 0.0018 μM); kinetics indicated that these were competitive inhibitors, with a *K*_i_ value of 0.0057 μM [112]. In 2024, the team further synthesized a group of *N*-arylated-4-yl-benzamides with slightly decreased activity featuring 2-aminothiazole-triazole bi-heterocycles (**2a**–**e**) (Figure 3). The 4-*N* ethyl group of the triazole ring was replaced by the phenyl group, and the original aromatic structure was extended to aryl entities with an amide (**2a**: IC_50_ = 1.277 μM; **2b**: IC_50_ = 0.371 μM; **2c**: IC_50_ = 0.419 μM; **2d**: IC_50_ = 0.025 μM). Meanwhile, the substitution of two methyl groups at the *ortho*-position enhanced the effective binding of the compound to the amino acids in the enzyme active site, compared to the substitution of other positions (**2e**: IC_50_ = 0.008 μM, *K*_i_ = 0.016 μM) [113].

By combining pharmacophores, the above group obtained indole-*N*-ethyltriazole hybrids (**3a**–**g**) (Figure 3) with excellent yields. *Mono*-substitution and di-substitution led to similar inhibitory activity. Compound **3a** (IC_50_ = 0.033 μM) exhibited the most significant inhibition of TYR in a non-competitive manner (*K*_i_ = 0.016 μM). For the single-substituted aryl group, *ortho*-substitution showed better activity than that at the *para-*position, and groups with reduced steric hindrance had improved access to the enzyme’s binding pocket (**3b**: IC_50_ = 0.036 μM; **3c**: IC_50_ = 0.076 μM). In terms of *para*-substituted compounds, **3d** (IC_50_ = 0.072 μM) was slightly better than **3c**, demonstrating that polar substituents may reduce the activity. Among the di-methylated regio-isomers, the level of molecular crowding significantly influenced the activity, with substitutions at the *ortho-* and *meta-*positions leading to a reduction in activity (**3e**: IC_50_ = 0.142 μM; **3f**: IC_50_ = 0.034 μM; **3g**: IC_50_ = 0.035 μM) [114].

Vanjare et al. successfully designed a series of (1,2,4-triazol-3-ylthio)-*N*-phenyl acetamide derivatives through bioisosterism (**4a**–**e**) (Figure 3). It was demonstrated that a reduction in the electronegativity of the halogen atom at the 4-position in the *N*-aryl functional group correlated with a more potent interaction among the chemical and the enzyme (**4a**: IC_50_ = 0.5425 μM; **4b**: IC_50_ = 0.1691 μM; **4c**: IC_50_ = 0.0605 μM). Moreover, it was observed that the IC_50_ values for **4d** and **4e** were 0.0238 μM and 0.2144 μM, respectively, illustrating that compounds with an aromatic group at the 4-position of the triazole ring exhibit a more favorable inhibitory effect compared to compounds with aliphatic substituents. Specifically, **4d** displayed 700-fold higher activity compared to the reference drug kojic acid (IC_50_ = 16.8320 µM). Additionally, a cytotoxicity assessment for **4d** utilizing the MTT assay on A375 human melanoma cells demonstrated its non-toxic properties within the effective range, indicating its potential as an excellent TYR inhibitor for further research [115].

Kloczkowski’s team synthesized several 1,2,4-triazole derivatives (**5a**–**d**) (Figure 3) through a multistep reaction pathway. The in vitro findings demonstrated a significant enhancement in the inhibitory efficacy of the derivatives as the number of fluorine atoms on both substituents increased. The IC_50_ values of **5a**, **5b**, **5c**, and **5d** were 0.219 μM, 0.124 μM, 0.111 μM, and 0.089 μM, respectively. Furthermore, **5d** demonstrated efficient binding to the TYR binding pocket and formed crucial hydrogen bonds with residue His-263. It could also be utilized as a promising chemical framework for the development of novel medications targeting melanogenesis and for future research [116].

Based on the potent inhibitory effect of kojic acid (KA) on TYR, Emami’s team employed a click reaction to conjugate natural products with potent TYR-inhibitory activity with KA via a triazole ring, resulting in the formation of a series of novel compounds (**6a**–**e**) (Figure 3). Notably, the inhibitory effects of these compounds were significantly enhanced with an increase in the number of oxygen atoms, facilitating the formation of effective hydrogen bonds with the amino acid residues within the binding pocket (**6a**: IC_50_ = 0.69 μM; **6b**: IC_50_ = 0.52 μM; **6c**: IC_50_ = 0.06 μM; **6d**: IC_50_ = 0.03 μM). Compound **6e** exhibited the best activity, with an IC_50_ value of 0.02 µM. The presence of conjugated acyl groups appears to heighten the inhibitory activity. Cell tests found that, within an effective range, the compounds obtained did not exhibit cytotoxicity [117].

**Figure 4:**
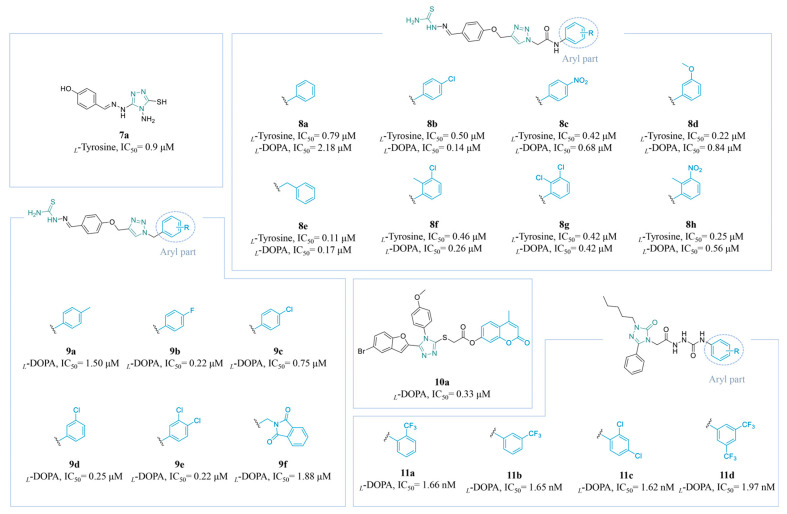
Chemical structures of TYR inhibitors **7a**–**11d** with potent anti-TYR activity.

Compound **7a** (IC_50_ = 0.9 µM) (Figure 4) was synthesized by Zhao’s team as a TYR inhibitor. Specifically, **7a** forms chelates with copper ions located in the active center of the enzyme and is specifically positioned with respect to its surrounding amino acids through *π–π* stacking and hydrogen bonding [118].

Hosseinpoor et al. synthesized a series of compounds (**8a**–**h**) (Figure 4) through the combination of aryl phenoxy methyl triazoles with thiosemicarbazides and subsequently evaluated their TYR-inhibitory activity. The inhibition of TYR was observed in all compounds. The in vitro activity experiments revealed that the number of substituents on the aryl group had no significant effect on the activity. Furthermore, it was noted that electron-donating groups (EDGs) displayed more significant monophenolase activity than electron-withdrawing groups (EWGs), whereas the diphenolase activity tended to decline. Notably, compound **8e** (*_L_*-DOPA: IC_50_ = 0.17 µM; *_L_*-tyrosine: IC_50_ = 0.11 µM), bearing a benzyl substitution, exhibited the most potent inhibitory activity against TYR [119].

In 2023, Divar et al. developed a novel series of substituted benzyl-triazole derivatives (**9a**–**f**) (Figure 4) linked to a hydrazinecarbothiamide scaffold, obtaining compounds similar to those of Hosseinpoor’s work. In contrast, compared to EDGs, the presence of *meta-* or *para-*EWGs on the benzyl moiety conferred superior inhibitory activity to the entire molecule, particularly compound **9b** (IC_50_ = 0.22 µM), with a *para*-fluoro group, and compound **9e** (IC_50_ = 0.22 µM), with a dichlorine substitution [120].

Coumarin derivatives are promising natural products with various biological effects. Bhat’s team employed molecular hybridization techniques to develop innovative coumarin-triazole hybrids. Compound **10a** (Figure 4) showed excellent anti-TYR activity and positive interactions with the core residues of TYR (**10a**: IC_50_ = 0.33 µM) [121].

Triazoles have demonstrated strong efficacy as TYR inhibitors. In Gultekin’s study, several new 1,2,4-triazole semicarbazide hybrid derivatives (**11a**–**d**) (Figure 4) were designed to inhibit TYR activity. The synthesized compounds demonstrated the potent inhibition of TYR at nanomolar concentrations (**11a**: IC_50_ = 1.66 nM; **11b**: IC_50_ = 1.65 nM, **11c**: IC_50_ = 1.62 nM, and **11d**: IC_50_ = 1.97 nM). These four derivatives show potential as effective candidates for the inhibition of TYR in pharmaceuticals or cosmetics [122].

**Figure 5:**
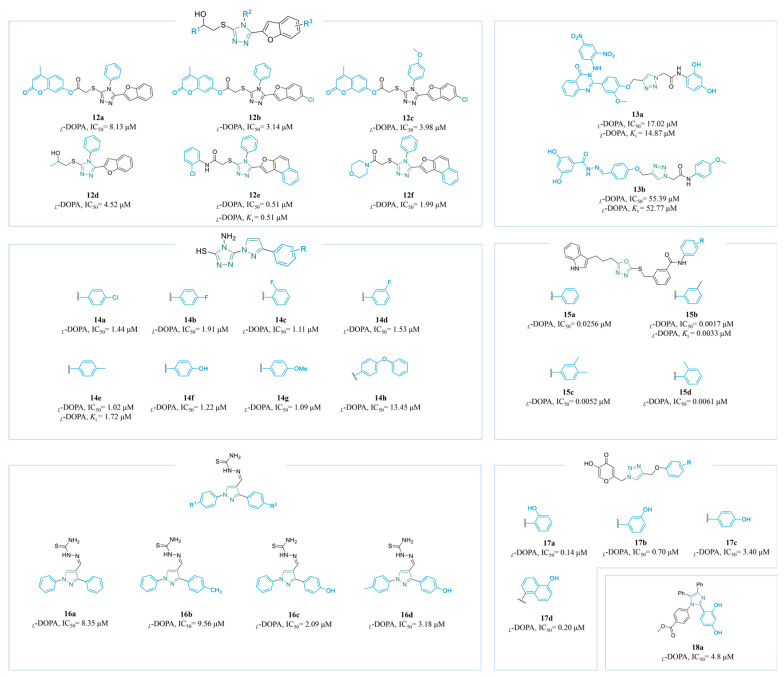
Chemical structures of TYR inhibitors **12a**–**18a** with potent anti-TYR activity.

Zahoo’s team synthesized a series of triazole derivatives: **12a**–**c** [121], **12d**–**e** [123], and **12f** [124] (Figure 5). Activity assessments indicated that the presence of electron-withdrawing groups on R^1^, in conjunction with the amide bond, significantly enhanced the biological activity. The *ortho*-chlorobenzene derivative, in particular, exhibited strong activity, with an IC_50_ value of 0.51 µM. Conversely, the phenyl ring on R^2^ was substituted with an electron-donating group in the *para*-position, leading to a slight reduction in activity (IC_50_ = 33.981 µM). Additionally, the incorporation of conjugated groups into the benzofuran ring was observed to further enhance the activity. Kinetic studies demonstrated that **12a** displayed noncompetitive inhibition, with a *K*_i_ of 0.07 mM. Furthermore, the molecular docking results indicated a docking score of −7.10 kcal/mol for **12a**, which was the lowest among the compounds examined.

Mahdavi’s group synthesized **13a**–**b** (Figure 5) by combining cinnamic acid [125] and a nitrophenylamino quinazolinone moiety [126] with triazole acetamide, separately. The 2,4-dinitrophenyl amine exhibited significantly enhanced activity, with the authors attributing its success to its interactions with multiple amino acid residues of TYR. Furthermore, **13a** served as a competitive inhibitor, with a *K*_i_ value of 14.87 µM.

Zhang et al. synthesized a series, **14a**–**h** (Figure 5), with significant inhibitory potential against TYR by combining pyrazole and triazole. Molecular docking studies revealed that the thiol group established a highly stable interaction with the Cu ion of TYR. Additionally, although the differences in electronegativity among the substituents on the benzene ring did not significantly influence the inhibition efficacy, the introduction of an additional phenyl group resulted in a rapid decrease in activity. This decline could be attributed to steric hindrance, interfering with the target molecule’s ability to effectively bind to the active site of TYR. In the meantime, they conducted toxicity assessments on **14e** (*K*_i_ = 1.72 µM) using MTT on human normal cells, demonstrating low toxicity across a concentration range of 0–90 μM, with the cell viability remaining above 80%. Notably, they also performed an anti-browning experiment on *Rosa roxburghii* Tratt fruit, which indicated that **14e** effectively inhibited the browning of the fruit [127].

A series of bi-heterocyclic benzamides, **15a**–**d** (Figure 5), have been reported by Nazir et al., all of which demonstrate exceptional activity. The effect of substituents on the acyl aromatic ring appears to be minimal regarding their activity. Notably, the meta-methyl-substituted **15b** (*K*_i_ = 0.0033 µM) exhibited the highest activity, while ortho-methyl and 2′,3′-dimethyl substitutions resulted in a slight reduction in activity. This decrease can be attributed to the presence of the methyl group, which interferes with the binding of the oxygen atom in the amide bond to the key amino acid residues in TYR [128].

Novel 1,3-diphenyl pyrazole-thiosemicarbazone compounds **16a**–**d** (Figure 5) have been synthesized by Azimi’s research group as potent inhibitors of TYR. The incorporation of the thiourea moiety significantly enhanced the binding affinity of these compounds for TYR, while the para-hydroxy group on the phenyl ring substantially increased their activity (**16c**: IC_50_ = 2.09 µM; **16d**: IC_50_ = 3.18 µM). Molecular docking studies indicated that the hydroxy group established additional hydrogen bonds with the critical active site residues, CYS83 and ASN81, of the enzyme. Kinetic analyses revealed that **16c** and **16d** inhibited TYR through a mixed inhibition mechanism. However, toxicological assessments showed that both compounds exhibited significant toxicity at a concentration of 8 µM, resulting in the cell viability levels dropping below 80% [129].

Ashooriha et al. utilized click chemistry to synthesize **17a**–**d** (Figure 5) by combining KA with phenolic structures. It was revealed that **17a** and **17c** demonstrated the highest potency in in vitro activity assays (**17a**: IC_50_ = 0.14 µM; **17c**: IC_50_ = 0.20 µM). Furthermore, antioxidant activity tests conducted on **17c** indicated an IC_50_ value of 10.1 µM, while toxicity assessments confirmed that it did not exhibit harmful effects on B16F10 cells at concentrations ranging from 2 to 8 µM. The significant anti-tyrosinase activity of **17c** is attributable to the interaction of its enolic head with multiple sites within the active site of TYR [130].

Utilizing a one-pot method, Rafique’s group synthesized the tetra-substituted imidazole derivative **18a** (Figure 5), which exhibited mixed-type inhibition (IC_50_ = 4.8 µM, *K*_i_ = 2.01 µM). The catechol structure significantly enhanced its inhibitory activity, primarily through interactions with the binding pocket, particularly around the hydroxyl site [131].

##### Thiazole Derivatives

**Figure 6:**
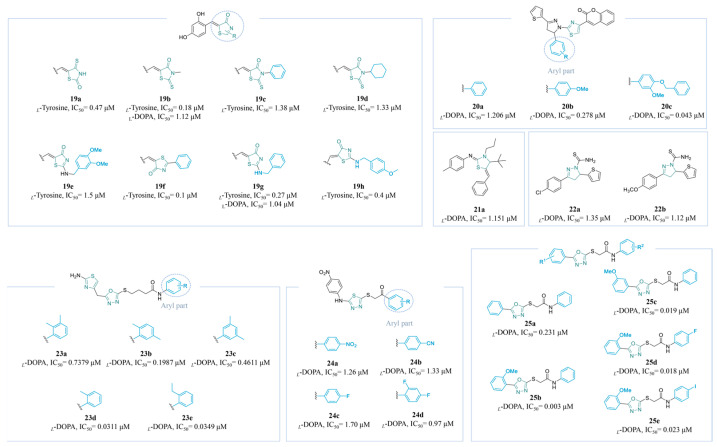
Chemical structures of TYR inhibitors **19a**–**25e** with potent anti-TYR activity.

Over the past 5 years, Moon’s team has been dedicated to the design of TYR inhibitors based on the thiazol-4 (5*H*)-one scaffold (**19a**–**g**) (Figure 6). Compounds **19a** [132], **19b** [133], **19c** [134], **19d** [135], **19e** [136], **19f** [4], **19g** [137], and **19h** [138] exhibited significantly potent inhibitory effects in respective structure series. Compound **19f** (IC_50_ = 0.1 µM), possessing a 2,4-dihydroxyl substituent, was a 190-fold more potent inhibitor than KA, displaying the best TYR inhibition. A B16F10 cell line evaluation demonstrated that **19f** led to a reduction in melanin production by inhibiting TYR and suppressing the expression of the TYR protein.

Hosseini Nasab et al. designed a group of novel thiophenyl-pyrazolylthiazole-coumarin compounds (**20a**–**c**) through a pharmacophore fusion strategy (Figure 6). It has been demonstrated that the incorporation of EDGs into the pyrazoline ring, including benzyloxy and methoxy, enhances the interaction with the active site of TYR (**20a**: IC_50_ = 1.206 µM; **20b**: IC_50_ = 0.278 μM; **20c**: IC_50_ = 0.043 μM) [139].

Compound **21a** (Figure 6) was synthesized using Zn^II^-catalysis in good yields and was proven to be an excellent TYR inhibitor, with an IC_50_ value of 1.151 µM [140]. Compounds **22a** and **22b** (Figure 6) were synthesized by Kisa’s laboratory. In vitro activity data showed that they were good inhibitors of TYR, with IC_50_ values of 1.35 µM and 1.12 µM, respectively [141].

A diverse series of bi-heterocyclic *N*-arylated butanamides were successfully synthesized using a convergent approach, resulting in the identification of new active scaffolds (**23a**–**e**) (Figure 6). Molecules with a single substituent demonstrated significantly higher inhibitory potential than those with two methyl groups or a slightly larger *ortho*-ethyl group, which experienced some steric hindrance. The IC_50_ values of **23a**–**c** are 0.7379 μM, 0.1987 μM, and 0.4611 μM, respectively, whereas those of **23d** and **23e** can be as high as 0.0311 μM and 0.0349 μM, respectively. Additionally, compound **23d**, featuring an *ortho*-methyl group on the aryl moiety, exhibits favorable access to the active site of TYR and establishes *π–π* interactions with His residues [142].

Ghani et al. conducted the synthesis of a series of thiadiazole derivatives (Figure 6) (Figure 5). Four compounds showed considerable inhibitory activity. Notably, **24d** (IC_50_ = 0.97 µM), bearing a difluorophenyl moiety at its terminal end, was identified as the most powerful TYR inhibitor [143].

A range of 1,3,4-oxadiazole scaffolds have been designed to inhibit TYR by Vanjare et al. (**25a**–**e**) (Figure 6). Derivative **25b**, a non-competitive inhibitor, exhibited remarkable activity among this series, with an IC_50_ = 0.003 µM, compared to **25a** (IC_50_ = 0.231 µM). The SARs of **25a**, **25b**, and **25c** (IC_50_ = 0.019 µM) showed that the *ortho*-methoxy phenyl group on the aryl part exhibited a better inhibitory activity pattern than the *meta*-methoxy phenyl group or un-substituted phenyl group. Interestingly, the substitution of halogen atoms did not yield a significant effect on the activity; docking studies confirmed these findings by producing consistent results (**25d**: IC_50_ = 0.018 µM; **25e**: IC_50_ = 0.023 µM) [144].

**Figure 7:**
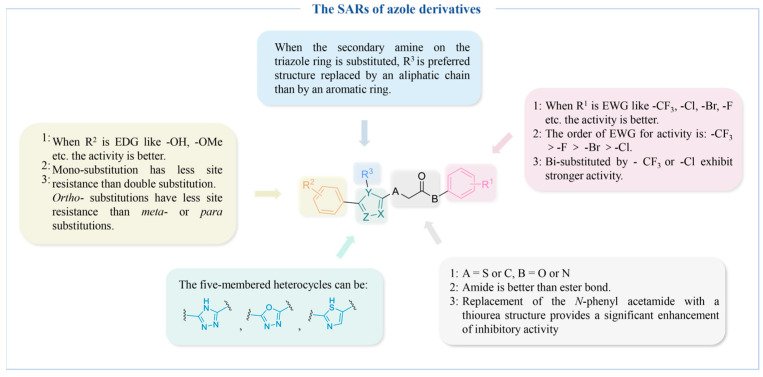
The SARs of azole-derived TYR inhibitors.

Figure 7 summarizes the SARs of the reviewed azole-derived TYR inhibitors.

#### 4.2.2. Thiourea Derivatives

**Figure 8:**
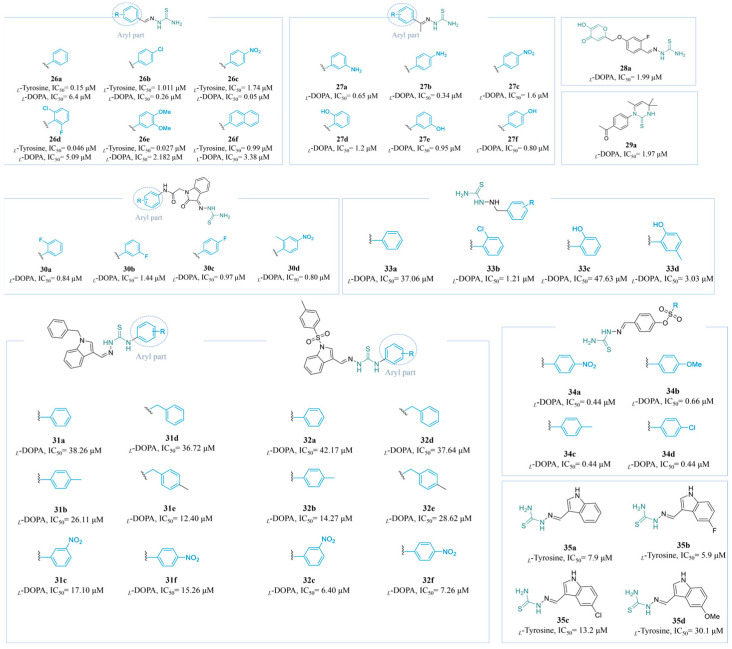
Chemical structures of TYR inhibitors **26a**–**35d** with potent anti-TYR activity.

Compounds containing a hydrazide structure exhibit a potent inhibitory effect on TYR. Khoshnevisadeh’s team developed highly potent TYR inhibitors derived from a thiosemicarbazide scaffold, resulting in 2-benzylidenehydrazine-1-carbothioamides (**26a**–**f**) (Figure 8). When *_L_*-DOPA was used as the substrate, **26c** (IC_50_ = 0.05 µM) with *para*-nitro was identified as the most efficient ligand. The EWGs at the *para-*positions of the benzyl moiety exhibited better activity than the EDGs. When using *_L_*-tyrosine as the substrate, **26e** exhibited the highest level of inhibition, with an IC_50_ value of 0.027 µM. Compounds with *meta*- and *para*-bis-methoxy substitutions exhibited approximately two-fold higher inhibitory efficacy compared to halogen binding. However, the introduction of a second aromatic moiety led to a notable reduction in inhibitory activity, likely attributable to its site-blocking effect, preventing effective binding to amino acid residues within the binding pocket [145].

Compounds **27b** (IC_50_ = 0.34 μM) and **27f** (IC_50_ = 0.80 μM) are novel competitive TYR inhibitors. The designed monosubstituted acetophenone thiosemicarbazone scaffolds were substituted with EDGs or EWGs at various positions on the benzene ring (**27a**–**g**) (Figure 8). EDGs are generally favored over EWGs, and substitution at the *para*-position demonstrates superiority over that at the *ortho*- and *meta*-positions. A docking study showed that the aminothiourea moiety enhanced the ability to penetrate the binding site of TYR, forming a hydrogen bond with the His-85 residue located near the catalytic center [146].

Peng et al. designed a series of hydroxypyranone-thiosemicarbazone derivatives through a structure conjunction strategy The derivative **28a** exhibited potent anti-TYR activity, with an IC_50_ value of 1.99 μM (Figure 8), being approximately 23 times more effective than KA. Thiosemicarbazone at the *para*-positions showed the best inhibitory activity, aligning with the presence of a confined hydrophobic pocket adjacent to the active site [147].

Compound **29a** (IC_50_ = 1.97 μM) (Figure 8) was synthesized and validated experimentally and theoretically as a potential inhibitor of TYR [148]. In previous research, Mahdavi’s team described the design and synthesis of a range of *N*-phenylacetamide-oxindole-thiosemicarbazide hybrids (**30a**–**d**) (Figure 8) as TYR inhibitors. In vitro tests demonstrated that all compounds were more potent than KA. The EWGs of the phenyl moiety exerted enhanced activity, e.g., **30a** bearing fluoro substituents at the *ortho*-position of the phenyl ring, **30c** featuring a fluorine substituent group at the *para*-position, and **30d** with methyl and nitro substituents on the phenyl ring at the *ortho*- and *para*-positions [149].

Shafiq and his colleagues investigated a series of indole-based thiosemicarbazone derivatives **31a**–**f** [150] and **32a**–**f** [151] (Figure 8). The results of in vitro activity studies indicated that, when the nitrogen atom in the indole ring was substituted with a benzyl group, the resultant compound with a benzyl substitution on the right-side aryl part demonstrated superior activity compared to its phenyl-substituted counterpart (**31b**: IC_50_ = 26.11 μM, **31e**: IC_50_ = 12.40 μM). Moreover, the presence of substituents on the phenyl group significantly enhanced the inhibitory activity, with **31e** exhibiting the highest potency. In contrast, when the nitrogen atom in the indole ring was substituted by tosyl, the efficacy of benzyl substitution on the aryl part was diminished. In this scenario, EWG substitutions enhance the activity more effectively than EDG substitutions, as evidenced by the greater potency of the *meta*-NO_2_ substitution compared to the *meta*-methyl substitution (**32c**: IC_50_ = 6.40 μM, *K*_i_ = 10.25 µM; **32b**: IC_50_ = 14.27 μM). Furthermore, meta substitutions generally outperform para substitutions (**32f**: IC_50_ = 7.26 μM).

Peng et al. reported the synthesis of a series of thiosemicarbazide derivatives **33a**–**d** (Figure 8). Among these derivatives, the *ortho*-chloro-substituted compound exhibited superior activity compared to the others, while the inhibitory effect of the compound with an EDG, **33c**, was significantly reduced (**33b**: IC_50_ = 1.21 μM; **33c**: IC_50_ = 47.63 μM). Notably, the introduction of a *para*-methyl group to **33c** alleviated the inhibitory effect of the hydroxyl group on TYR (**33d**: IC_50_ = 3.03 μM). Furthermore, anti-browning assay results demonstrated that **33b** effectively inhibited the browning of fresh apple juice. Meanwhile, **33b** showed no cytotoxic effects on human HEK-293 cells across a concentration range of 3 to 96 μM, suggesting that it is a promising candidate for use as a tyrosinase inhibitor [152].

Pivetta’s group recently modified (*E*)-2-(4-hydroxybenzylidene)hydrazine-1-carbothioamide to synthesize **34a**–**d** (IC_50_ range: 0.44~0.66 μM) (Figure 8), which all exhibited greater activity than the positive control, KA (IC_50_ = 18 μM). The influence of the electronegativity of the substituents in the para-position on the biological activity was found to be insignificant. Furthermore, no compounds displayed cytotoxicity towards the HaCaT cell line of human keratinocytes within an effective concentration range of 0.5 to 50 μM [153].

Xu et al. synthesized a series of indole-thiourea derivatives, **35a**–**d** (Figure 8). The experimental results indicated that **35d**, which incorporated an EDG, exhibited reduced inhibitory activity (IC_50_ = 30.1 μM). In contrast, halogen substituents, which possess electron-withdrawing characteristics, significantly enhanced the anti-TYR activity of **35c** and **35d** (**35c**: IC_50_ = 5.9 μM; **35d**: IC_50_ = 13.2 μM). Furthermore, the competitive inhibition of **35b** was confirmed through Lineweaver–Burk plots [154].

**Figure 9:**
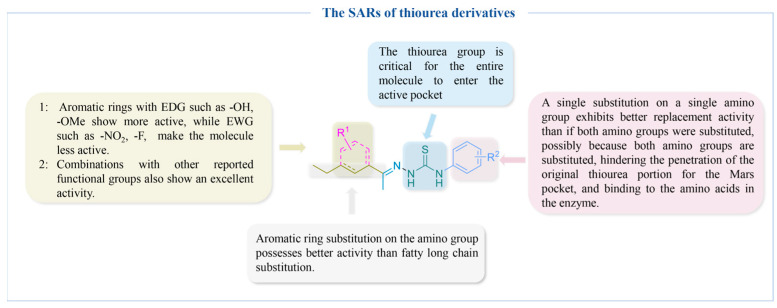
The SARs of thiourea-derived TYR inhibitors.

Figure 9 summarizes the SARs of the reviewed thiourea-derived TYR inhibitors.

#### 4.2.3. Amide and Thioamide Derivatives

**Figure 10:**
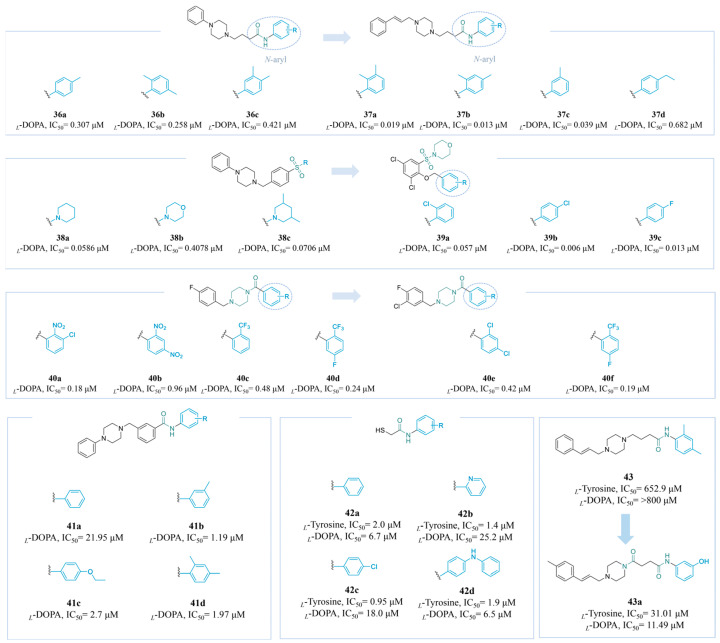
Chemical structures of TYR inhibitors **36a**–**43a** with potent anti-TYR activity.

Seo’s team designed and synthesized a set of *N*-(substituted-phenyl)-4-(4-phenyl-1-piperazinyl) butanamides (**36a**–**c**) [155] and *N*-(substituted-phenyl)-4-((4-[(*E*)-3-phenyl-2-propenyl]-1-piperazinyl)) butanamides (**37a**–**d**) (Figure 10) [156]. The in vitro inhibition study of mTYR showed that all compounds from both scaffolds were excellent inhibitors. In particular, derivatives **36b** (IC_50_ = 0.258 μM) and **37b** (IC_50_ = 0.013 μM) emerged as the most potent compounds relative to KA (IC_50_ = 16.841 μM). The compound featuring a lengthy three-carbon chain bridging the benzene ring and the piperazine ring exhibited better activity. SARs revealed a significant impact on TYR inhibition with the presence of two methyl groups in the *N*-aryl moiety for both scaffolds, demonstrating stronger effects compared to a *mono*-substituent. Moreover, **37d**, with a bulky ethyl group, showed relatively lower inhibitory potential in the synthetic series, suggesting that the presence of less bulky substituents in the *N*-aryl part may have facilitated strong interactions and the effective occupation of the enzyme.

In addition to the above structures, this research group also designed and synthesized sulfonamide TYR inhibitors (**38a**–**c**) [157] and (**39a**–**c**) [158] with heterocyclic rings (Figure 10). For **38a**–**c**, it could be elucidated that compounds with a substituted piperidinyl ring possessed slightly lower inhibitory potential as compared to the un-substituted piperidinyl ring. The presence of a methyl group in this heterocyclic ring may potentially result in steric repulsion, leading to a slight reduction in interactions with the enzyme. For a 4-((3,5-dichloro-2-((2/4-halobenzyl) oxy) phenyl) sulfonyl) morpholine scaffold, the benzyl group substituted in the *para-*position is more advantageous than at the *ortho-*position. Compounds with *para*-*Cl* exhibited even higher activity compared to the derivatives with a *para*-F group, which was presumably attributed to the increased polarizability of the -*Cl* group.

Luca’s team investigated the potential of 4-(4-fluorobenzyl) piperazin-1-yl)-based derivatives (Figure 10) as TYR inhibitors [159]. Among the compounds characterized by the presence of EDG substituents, derivatives with an amino substitution showed excellent inhibitory activity, e.g., **40a** with an IC_50_ value of 0.18 μM (Figure 10). Then, the team integrated the 3-chloro-4-fluorophenyl fragment into different chemical structures (**40e**–**f**) (Figure 10) [160]. However, this strategy did not significantly impact the TYR inhibition.

Zeb et al. synthesized a series of *N*-(aryl)-3-[(4-phenyl-1-piperazinyl)methyl]benzamides **41a**–**d** (Figure 10) and evaluated their inhibitory potency. Compounds featuring EDGs on the aromatic ring exhibited enhanced activity relative to those lacking substituents. Notably, the *meta*-substituted methyl compound **41b** displayed the most significant inhibitory effect, with an IC_50_ value of 1.19 μM, in a competitive type of kinetic mechanism [161].

Moon’s laboratory devised and synthesized 2-mercapto-*N*-arylacetamide analogs **42a**–**d** (Figure 10) to identify novel TYR inhibitors. Notably, **42c**, featuring a *para*-Cl substitution in the aromatic portion, exhibited the strongest monophenolase-inhibitory effect, with an IC_50_ value of 0.95 μM. Meanwhile, **42d**, substituted with -*NHPh*, displayed the most potent diphenolase-inhibitory activity (*_L_*-DOPA, IC_50_ = 6.5 μM). Substitutions with EWGs were found to enhance the monophenolase activity while reducing the diphenolase activity. However, the incorporation of bulky phenylamino substitutions had a negligible impact on diphenolase inhibition. The derivatives **42b**, **42c**, and **42d** were characterized as competitive inhibitors, exhibiting *K*_i_ values of 47.5, 18.5, and 12.8 μM, respectively. In vitro experiments conducted with B16F10 cells demonstrated that **42a**–**d** possessed significant anti-melanogenic effects. Furthermore, in vivo studies using zebrafish embryos confirmed their high efficacy [162].

Recent advancements in artificial intelligence have enabled Bai’s group to utilize AI de novo molecular generation in synthesizing **43a** (Figure 10). This compound demonstrated effects comparable to those of KA (*_L_*-tyrosine, IC_50_ = 38.55 μM; *_L_*-DOPA, IC_50_ = 17.32 μM), with IC_50_ values of 31.01 μM for *_L_*-tyrosine and 11.49 μM for *_L_*-DOPA, reflecting an approximately 60-fold increase in activity compared to the lead compounds. Moreover, **43a** exhibited significant anti-pigmentation activity in a zebrafish model. Metabolic stability studies further revealed its susceptibility to hepatic metabolism [163].

**Figure 11:**
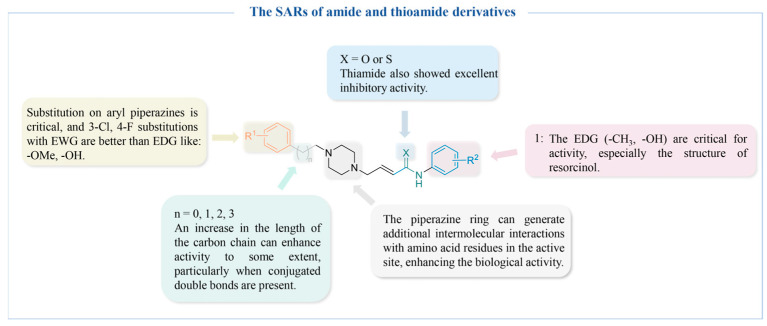
The SARs of amide- and thioamide-derived TYR inhibitors.

Figure 11 summarizes the SARs of the reviewed amide- and thioamide-derived TYR inhibitors.

#### 4.2.4. Cinnamic Acid Derivatives

**Figure 12:**
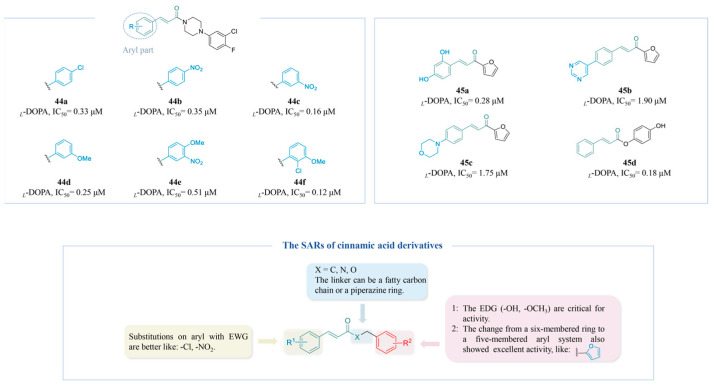
Chemical structures of TYR inhibitors **44a**–**45d** with potent anti-TYR activity and the SARs of cinnamic acid-derived TYR inhibitors.

Applying structure-based drug design, Romagnoli et al. designed a novel series of cinnamides comprising derivatives of cinnamic acid (**44a**–**f**) (Figure 12) [164]. It could be inferred through the docking study that the remarkable inhibitory activity against TYR was attributed to the presence of the arylpiperazine motif. Moreover, the activity was less affected by the nature and position of the substituents on the aryl of the cinnamoyl moiety. However, results from other studies further suggest that altering the substituent group on the aryl can yield diverse outcomes, as evidenced by **45a** (IC_50_ = 0.28 μM) [165], **45b** (IC_50_ = 1.90 μM), **45c** (IC_50_ = 1.75 μM) [166], and **45d** (IC_50_ = 0.18 μM) (Figure 12) [167], challenging the initial conclusions. These results suggest that the activity of the cinnamoyl moiety may be significantly influenced by the type and location of substituents on the aryl group. Additionally, incorporating structures based on resorcinol and hydroquinone has the potential to significantly enhance the TYR activity.

Figure 12 summarizes the SARs of the reviewed cinnamic acid-derived TYR inhibitors.

#### 4.2.5. Benzo Five-Membered Heterocyclic Derivatives

**Figure 13:**
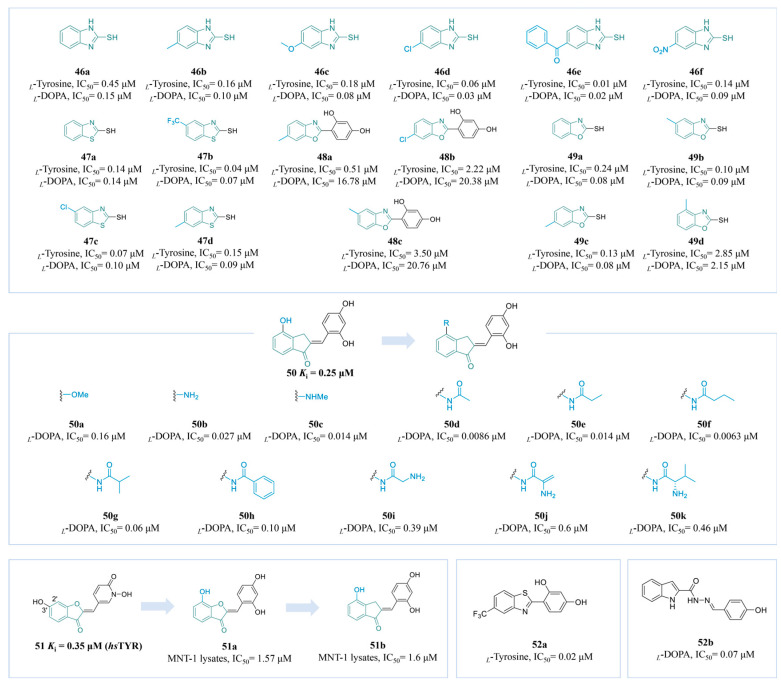
Chemical structures of TYR inhibitors **46a**–**52b** with potent anti-TYR activity.

Compounds containing sulfhydryl groups can bind copper ions at the active site of TYR, leading to the inhibition of enzyme activity. Moon’s research group synthesized ten 2-mercaptobenzimidazole (2-MBI) analogs (**46a**–**f**) (Figure 13) and evaluated their activity. Compound **46e** (*_L_*-tyrosine, IC_50_ = 0.01 µM; *_L_*-DOPA, IC_50_ = 0.02 µM), with a 5-benzoyl substituent, was a potent inhibitor. SARs showed that the substitution at position 5 on 2-MBI had a greater effect on the activity than that at other positions, while EWGs displayed better inhibitory activity compared to EDGs [168]. In 2024, the research group conducted in vitro cell viability assays and in vivo melanogenesis inhibition tests using zebrafish to evaluate these compounds. The findings revealed that none of the compounds exhibited toxicity at concentrations below 20 µM. Furthermore, the zebrafish studies demonstrated that compound 4, which contained an EWG, displayed notable decolorization effects, thereby reinforcing its capacity to inhibit TYR [169].

The above team reported four 2-thiobenzothiazole derivatives (**47a**–**d**) (Figure 13). Compound **47b**, characterized by an EWG, demonstrated a significant inhibitory effect on TYR at very low concentrations (*_L_*-tyrosine, IC_50_ = 0.04 μM, *_L_*-DOPA, IC_50_ = 0.07 μM). Additionally, none of the examined compounds exhibited significant toxicity at concentrations below 20 µM, with the effective inhibition of melanin synthesis in B16F10 cells observed at 10 µM. In vivo tests using zebrafish embryos showed similar results [170]. Inspired by the tyrosinase-inhibitory activity of compounds with a 2-phenylbenzo[*d*]thiazole scaffold, the group explored phenolic compounds **48a**–**c** (Figure 13) with 2-phenylbenzo[*d*]oxazole derivatives as novel tyrosinase inhibitors. The resorcinol group enhanced the activity of its derivatives, with the *para*-methyl-substituted compound **48a** recognized as the most effective inhibitor (*_L_*-tyrosine, IC_50_ = 0.51 μM, *_L_*-DOPA, IC_50_ = 16.78 μM). Kinetic studies suggest that it functions as a mixed-type inhibitor. Furthermore, at a concentration of 5 μM, it demonstrates no toxicity towards either B16F10 or HaCaT cells. These findings highlight its potential for development as a TYR inhibitor [171]. 2-Mercaptobenzoxazole analogs **49a**–**d** (Figure 13) were also synthesized in 2024. Among them, the compound **49c**, which possesses a 3′-methyl substitution on the benzene ring, exhibits excellent TYR inhibition activity, with an IC_50_ value of 0.13 μM for *_L_*-tyrosine and an IC_50_ value of 0.08 μM for *_L_*-DOPA. Additionally, it shows no toxicity towards cells at a concentration of 20 μM [172].

Compound **50** is recognized as one of the most active derivatives reported thus far for the inhibition of TYR. Expanding on this lead structure, Lazinski’s team investigated the potential of various groups introduced to the 4-position (**50a**–**k**) (Figure 13). All compounds listed showed excellent activity, particularly linear aliphatic amides, showing superior efficacy. Compounds **50d** and **50f** with acetyl and propionyl displayed the lowest inhibitory potency (**50d**: IC_50_ = 0.0086 μM; **50f**: IC_50_ = 0.0063 μM). The molecular docking structure suggests that the amino group at the 4-position binds to the TYR with a hydrogen bond and is critical for the inhibitory activity [173].

Compound **51** is recognized as one of the most effective human TYR inhibitors reported to date (*K*_i_ = 0.35 μM) (Figure 13). However, its permeability is suboptimal. To enhance this characteristic, Roulier and his colleagues replaced the original inadequate scaffold with a dihydroxybenzene structure, resulting in compound **51a**, which exhibited an IC_50_ value of 1.57 μM. This compound demonstrated remarkable efficacy in assays utilizing human melanoma cell lysates, with the hydroxyl group at the 2′ position of the benzene ring playing a significant role in the inhibition of melanin production [27].

Compounds **52a** [174] and **52b** [175] (Figure 13) were also reported as potent TYR inhibitors, with IC_50_ values of 0.2 μM and 0.07 μM, respectively.

**Figure 14:**
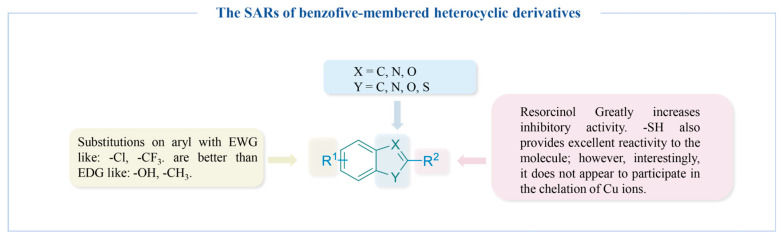
The SARs of benzo-five-membered heterocyclic-derived TYR inhibitors.

Figure 14 summarizes the SARs of the reviewed benzo-five-membered heterocyclic-derived TYR inhibitors.

#### 4.2.6. Other Derivatives

**Figure 15:**
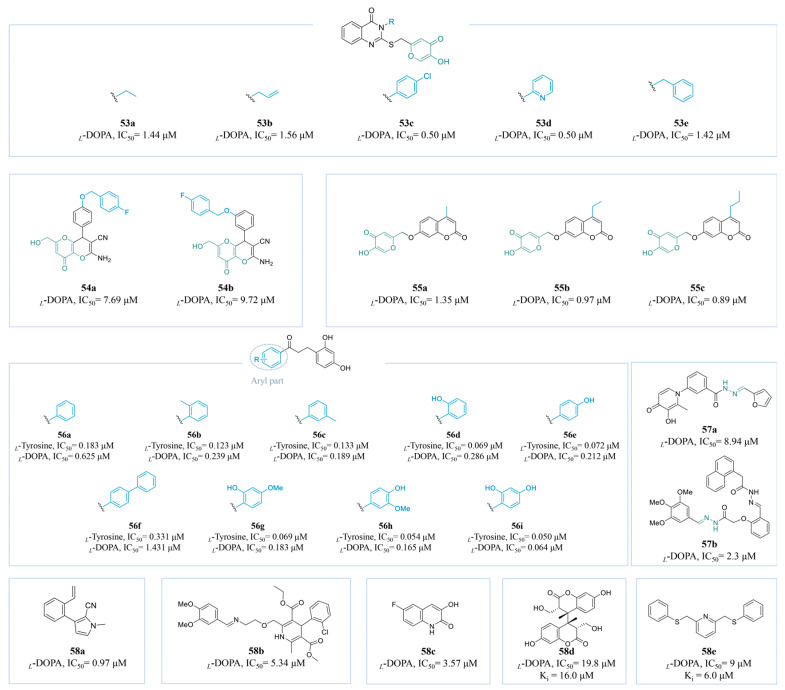
Chemical structures of TYR inhibitors **53a**–**58e** with potent anti-TYR activity.

Sepehri et al. identified a range of compounds containing kojyl thioether linked to various quinazoline derivatives (**53a**–**e**) (Figure 15). An SAR study showed that an aryl substitution on the quinoline ring led to better activity than aliphatic chain substitution. EWGs on aryl groups showed superior activity, exemplified by a *Cl*-containing derivative with moderate lipid solubility (**53c**, IC_50_ = 0.50 µM). Similarly, the pyridine derivative **53d** demonstrated significant inhibitory potential, with an IC_50_ of 0.50 µM [176].

A new series of KA-fused 2-amino-3-cyano-4*H*-pyran derivatives was designed by Najaf et al. through the integration of KA, benzyloxy benzaldehyde, and malononitrile. In vitro activity assays revealed that the *para*-substituted compound **54a** (IC_50_ = 7.69 µM, *K*_i_ = 7.57 μM) demonstrated superior inhibitory effects compared to the *meta*-substituted compound **54b** (IC_50_ = 9.72 µM) (Figure 15). Importantly, although **54a** was synthesized as a racemate, the *R*-enantiomer exhibited significantly stronger interactions than the *S*-enantiomer [177].

He and his colleagues synthesized a series of compounds **55a**–**c** (Figure 15) by combining KA and coumarin. As the length of the lipid chain substituent on coumarin increased, the biological activity was consistently enhanced. Molecular docking analyses revealed that the elongation of the carbon chain altered the hydrophobic environment of the amino acid residues at the active site of TYR, resulting in the inhibition of TYR activity (**55c**: IC_50_ = 0.89 μM, *K*_i_ = 3.54 µM). Meanwhile, toxicity assessments conducted using the human normal cell line HEK-293 demonstrated that the cell viability remained above 80% within a concentration range of 4 μM to 108 μM, thereby confirming the safety of the compounds [178].

In previous research, Xue et al. successfully synthesized a series of potent TYR inhibitor hybrids through the fusion of a dihydrochalcone backbone with a resorcinol structural pharmacophore (**56a**–**i**) (Figure 15). Compound **57i** (*_L_*-tyrosine, IC_50_ = 0.050 µM; *_L_*-DOPA, IC_50_ = 0.064 µM) exhibited the most potent inhibitory activity. Compounds in which a methyl group replaced one of the hydroxyl groups (**56i** with 2,4-dihydroxyphenyl; **56h** with 3-methoxy-4-hydroxyphenyl) also exhibited remarkable inhibitory effects, while the presence of large site-blocking groups hindered their access to the active site, resulting in a reduction in overall activity. Notably, **56i** showed excellent performance in an in vitro skin penetration test, with low cytotoxicity [179].

*Bis-*Schiff base derivatives have recently garnered significant attention in the field of tyrosinase inhibitors, with two research groups successfully synthesizing effective variants. Compounds **57a** [180] and **57b** [181] (Figure 15) exhibit remarkable IC_50_ values of 8.94 μM and 2.3 μM, respectively.

Compounds **58a** [182], **58b** [183], **58c** [184], **58d** [185], and **58e** [186] (Figure 15) were also reported as potent TYR inhibitors, with IC_50_ values of 0.97 μM, 0.2 μM, and 0.07 μM, respectively. Nitrogen-containing heterocycles possessing a phenolic structure show better inhibitory activity.

## 5. Conclusions

Melanin is important to protect the skin from UV rays. However, excessive pigmentation leads to freckles, chloasma, age spots, sun spots, and other conditions and even results in melanoma. Hence, regulating melanin production is pivotal in the treatment of hyperpigmentation. A range of anti-melanogenic agents have been developed to inhibit tyrosinase, facilitate melanosome maturation, and influence transport processes, alongside other signaling pathways related to melanin synthesis [187]. Most importantly, due to the critical role of TYR in melanin biosynthesis, it has become a key target in the management of hyperpigmentation.

This review provided a detailed overview of TYR and its structural features and catalytic mechanism in melanin biosynthesis. Moreover, we conducted a comprehensive analysis of the latest research advances in small-molecule TYR inhibitors. The SARs of the chemical series of representative TYR inhibitors were also summarized. In conclusion, compounds synthesized through artificial methods typically exhibit more potent inhibitory activity compared to their natural counterparts. Additionally, the incorporation of artificial intelligence has significantly advanced drug design processes, enabling the rapid identification of small-molecule compounds with improved efficacy and favorable drug-like properties [163]. Notably, this relevant content will provide important practical information for those engaged in anti-pigmentation research, especially the design and discovery of efficient TYR inhibitors.

## Figures and Tables

**Table 1:** Natural polyphenol-based TYR inhibitors reported from 2015 to the present.

Compound	Source	Chemical Structure	Inhibition Mechanism	TYR Inhibition (IC_50_) ^a^		Ref.
				*_L_*-Tyrosine	*_L_*-DOPA	
**Polyphenols**						
Sesamol (**1**)	*Sesamum indicum*	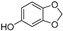	-	-	0.6 μM	[72]
*N*-Acetyldopamine (**2**)	*Protaetia brevitarsis seulensis*	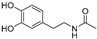	-	-	44.8 μM	[73]
(**3**)	*Wedelia trilobata*	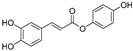	-	-	2.0 μM	[74]
6′-*O*-Caffeoylarbutin (**4**)	Quezui Tea	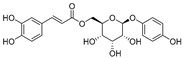	Competitive	1.1 μM	>50 μM	[75]
Tamariscinol U (**5**)	*Selaginella tamariscina*	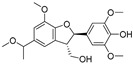	-	-	5.8 μM	[76]
(**6**)	*Carica* *papaya*	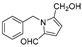	-	25.5 μM	-	[77]
Lariciresinol (**7**)	*Carica* *papaya*	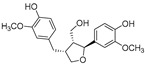	-	19.8 μM	-	[77]
(**8**)	*Symphyocladia latiuscula*	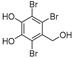	Competitive	10.8 μM	>50 μM	[78]
(**9**)	*Symphyocladia latiuscula*	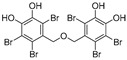	Competitive	2.9 μM	>50 μM	[78]
Puerol A (**10**)	*Amorpha fruticosa*	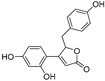	Competitive	2.2 μM	3.9 μM	[68]
**Phenolic acids**						
*p*-Coumaric acid (**11**)	*Lepechinia meyenii*	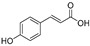	Non-competitive	0.3 μM	0.6 μM	[79]
Caffeic acid (**12**)	*Lepechinia meyenii*	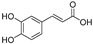	Non-competitive	1.5 μM	2.3 μM	[79]
Rosmarinic acid (**13**)	*Lepechinia meyenii*	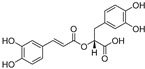	Non-competitive	4.1 μM	8.6 μM	[79,80]
Caftaric acid (**14**)	*Lepechinia meyenii*	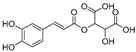	Competitive	-	30.0 μM	[81]
Ascorbic acid (**15**)	Fuji apple	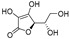	-	-	13.4 μM	[82]
(**16**)	*Protea cynaroides*	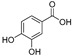	Competitive	0.88 μg/mL	-	[83]
(**17**)	*Protea cynaroides*	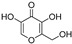	Competitive	0.72 μg/mL	-	[83]
**Flavonoids—flavones**						
Luteolin (**18**)	*Perilla* seeds	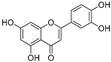	-	-	24.6 μM	[80]
Apigenin (**19**)	*Perilla* seeds	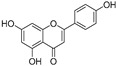	-	-	49.3 μM	[80]
Chrysoeriol (**20**)	*Perilla* seeds	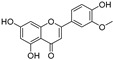	-	-	35.8 μM	[80]
(**21**)	Petals and foliage	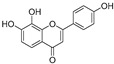	Non-competitive	-	10.3 μM	[84]
Panicolin (**22**)	Petals and foliage	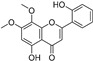	Competitive	-	2.75 μg/mL	[85]
Broussoflavonol H (**23**)	*Broussonetia papyrifera*	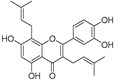	-	13.7 μM	-	[86]
Norartocarpetin (**24**)	*Artocarpus rigida*	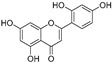	-	0.02 μM	-	[87]
**Flavonoids—flavanones**						
Artocarpanone (**25**)	*Artocarpus heterophyllous*	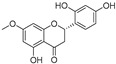	-	--	2.0 μM	[88]
Liquiritigenin (**26**)	*Artocarpus heterophyllous*	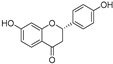	-	-	22.0 μM	[88]
Steppogenin (**27**)	*Artocarpus heterophyllous*	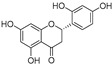	-	-	7.5 μM	[88]
Kushenol A (**28**)	*Sophora flavescens*	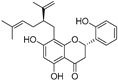	Non-competitive	1.1 μM	-	[89]
Kurarinone (**29**)	*Sophora flavescens*	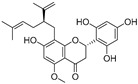	Mixed	7.1 μM	-	[90]
Sophoraflavanone G (**30**)	*Sophora flavescens*	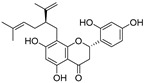	Mixed	66.7 μM	-	[90]
6-Prenylanringenin (**31**)	*Humulus lupulus*	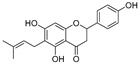	Mixed	38.1 μM	>50 μM	[91]
**Flavonoids—flavonols**						
8-Prenylkaempferol (**32**)	*Sophora flavescens*	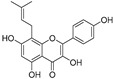	Competitive	2.4 μM	-	[89]
Kushenol (**33**)	*Sophora flavescens*	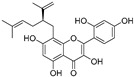	Non-competitive	24.1 μM	-	[89]
Lsoanhydroicaritin (**34**)	*Sophora flavescens*	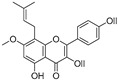	Mixed	0.7 μM	-	[90]
Quercetin (**35**)	Rose flowers	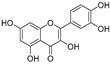	Competitive	4.2 μM	10.7 μM	[92,93]
Kaempferol (**36**)	Rose flowers	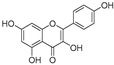	Competitive	5.5 μM	-	[92]
Galangin (**37**)	*Alpinia officinarum*	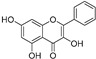	Competitive	-	3.6 μM	[71]
Broussoflavonol I (**38**)	*Broussonetia papyrifera*	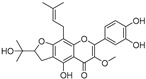	-	29.6 μM	-	[86]
Broussoflavonol K (**39**)	*Broussonetia papyrifera*	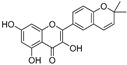	-	17.6 μM	-	[86]
Glycyrrhiza flavonol A (**40**)	*Broussonetia papyrifera*	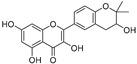	-	20.7 μM	-	[86]
Papyriflavonol A (**41**)	*Broussonetia papyrifera*	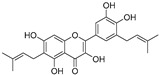	-	29.6 μM	-	[86]
Broussoflavonol F (**42**)	*Broussonetia papyrifera*	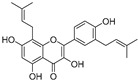	-	29.7 μM	-	[86]
Broussoflavonol B (**43**)	*Broussonetia papyrifera*	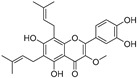	-	31.7 μM	-	[86]
Isolicofavonol (**44**)	*Broussonetia papyrifera*	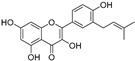	-	24.7 μM	-	[86]
**Flavonoids—flavanonols**						
*Trans*-dihydromorin (**45**)	*Morus alba*	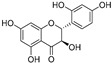	-	-	9.4 μM	[94]
Broussoflavonol J (**46**)	*Broussonetia papyrifera*	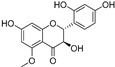	-	-	9.3 μM	[86]
**Flavonoids—isoflavones**						
Formononetin (**47**)	*Sophora flavescens*	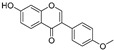	Non-competitive	19.9 μM	-	[89]
(**48**)	*Pichia pastoris*	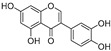	Competitive	-	15.9 μM	[95]
Daidzein (**49**)	*Pueraria lobata*	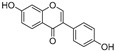	-	-	17.5 μM	[96]
Lupinalbin A (**50**)	*Apios americana*	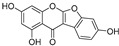	Competitive	-	10.3 μM	[97]
Calycosin (**51**)	*Pueraria lobata*	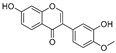	Competitive	1.5 μM	7.0 μM	[98]
Semilicoisoflavone B (**52**)	*Glycyrrhiza inflata*	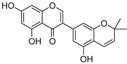	-	-	0.3 μM	[99]
Allolicoisoflavone B (**53**)	*Glycyrrhiza inflata*	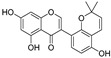	-	-	0.8 μM	[99]
**Flavonoids—aurones**						
(**54**)	*Morus notabilis*	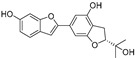	Competitive	-	14.8 μM	[100]
Moracin M (**55**)	*Morus alba* L.	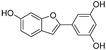	-	8.0 μM	-	[101]
Moracin B (**56**)	*Morus alba* L.	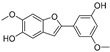	--	34.4 μM	-	[101]
Moracin VN (**57**)	*Artocarpus heterophyllus*	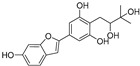	Non-competitive	-	0.8 μM	[102]
**Flavonoids—chalcones**						
Isoliquiritigenin (**58**)	*Pueraria lobata*	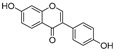	-	-	4.9 μM	[96]
Xanthohumol (**59**)	*Humulus lupulus*	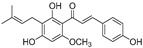	Competitive	15.4 μM	31.1 μM	[91]
(**60**)	*Humulus lupulus*	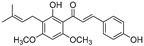	Competitive	34.3 μM	>50 μM	[91]
Xanthohumol C (**61**)	*Humulus lupulus*	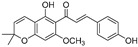	Competitive	20.6 μM	41.3 μM	[91]
Xanthoumol B (**62**)	*Humulus lupulus*	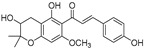	Competitive	22.1 μM	46.7 μM	[91]
(**63**)	*Morus alba* L.	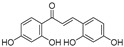	-	0.07 μM	-	[101]
**Flavonoids—anthocyanidins**						
Cyanidin (**64**)	*Diospyros kaki*	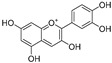	Competitive	-	9.1 μM	[103]
Luteolinidin (**65**)	*Sorghum bicolor*	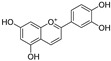	Competitive	-	3.7 μM	[104]
**Stilbenes**						
Oxyresveratrol (**66**)	*Morus alba*	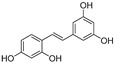	-	-	1.7 μM	[94]
(**67**)	*Morus alba*	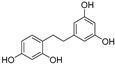	-	-	0.3 μM	[94]
(**68**)	*Morus alba*	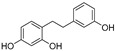	-	-	0.8 μM	[94]
Caricapapayol (**69**)	*Carica papaya*	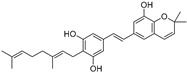	-	14.3 μM	-	[77]
**Flavonolignans**						
Isosilybin A (**70**)	*Silybum marianum*	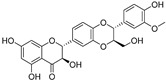	Mixed	2.1 μM	16.7 μM	[105]
Isosilybin B (**71**)	*Silybum marianum*	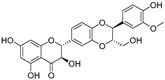	Mixed	4.9 μM	19.8 μM	[105]
(**72**)	*Silybum marianum*	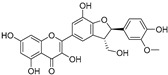	Mixed	7.6 μM	35.9 μM	[105]
Silychristin A (**73**)	*Silybum marianum*	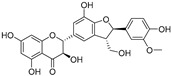	Mixed	3.2 μM	28.8 μM	[105]
Silychristin B (**74**)	*Silybum marianum*	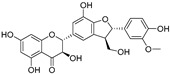	Mixed	4.5 μM	44.9 μM	[105]
**Other natural products—polyphenols**						
Neorauflavane (**75**)	*Campylotropis hirtella*	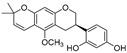	Competitive	0.03 μM	0.5 μM	[106]
trans-*N*-Coumaroyltyramine (**76**)	*Humulus japonicus*	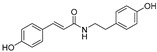	-	-	40.6 μM	[107]
Caffeine (**77**)	*Camellia pollen*	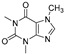	Non-competitive	18.6 μg/mL	-	[108]
Arichostatin A (**78**)	*Streptomyces* sp.	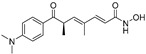	Mixed	-	2.3 μM	[109]
Deoxytrichostatin A (**79**)	*Streptomyces* sp.	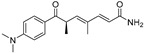	-	-	19.2 μM	[109]
10-Methoxy-leonurine (**80**)	*Leonurus japonicas*	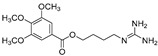	Competitive	7.4 μM	-	[110]
Leonurine (**81**)	*Leonurus japonicas*	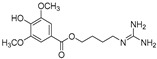	Competitive	12.4 μM	-	[110]
Emodin (**82**)	*Leonurus japonicas*	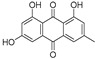	-	-	29.0 μM	[110]
Physcion (**83**)	*Leonurus japonicas*	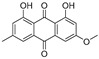	-	-	32.0μM	[111]

^“a”^: mushroom TYR. “-”: not reported.

## Data Availability

Not applicable.

## Abbreviations

*α*-MSH, *α*-melanocyte-stimulating hormone; cAMP, cyclic adenosine monophosphate; DHI, 5,6-dihydroxyindole; DHICA, 5,6-dihydroxyindole carboxylic acid; DQ, dopaquinone; EDGs, electron-donating groups; *E_deoxy_*, the deoxy form; *E_met_*, the methoxy form; *E_met_*D, diphenolase E_met_ complex; *E_met_*M, inactive complex; *E_oxy_*, the oxygen form; *E_oxy_*M, monophenolase *E_oxy_* complex; EWGs, electron-withdrawing groups; HQ, hydroquinone; IL-2, interleukin-2; KA, kojic acid; *_L_*-DOPA, *_L_*-dihydroxyphenylalanine; MAPK, mitogen-activated protein kinase; MC-1R, melanocortin 1 receptor; MITF, microphthalmia-associated transcription factor; mTYR, *Agaricus bisporus* TYR; NO, nitric oxide; PI3K, phosphatidylinositol-3-kinase; PKA, protein kinase A; Ref, reference; SAR, structure–activity relationship; SKMEL-188, Sanger Institute’s Human Melanoma Cell Line 188; TYR, tyrosinase; TYRP-1, TYR-related protein 1; TYRP-2, TYR-related protein 2; UVR, ultraviolet radiation.
